# In vitro impact preliminary assessment of airborne particulate from metalworking and woodworking industries

**DOI:** 10.1038/s41598-021-99815-7

**Published:** 2021-10-12

**Authors:** Ilona Pavlovska, Anna Ramata-Stunda, Zanna Martinsone, Martins Boroduskis, Liene Patetko, Inese Martinsone, Anita Seile, Ivars Vanadzins

**Affiliations:** 1 Laboratory of Hygiene and Occupational Diseases, Institute of Occupation Safety and Environmental Health, Riga Stradins University, Riga, Latvia; 2 Laboratory of Bioanalytical and Biodosimetry Methods, Faculty of Biology, University of Latvia, Riga, Latvia

**Keywords:** Cell biology, Health occupations, Risk factors

## Abstract

Inhalation is the main route of exposure to airborne pollutants. To evaluate the safety and assess the risks of occupational hazards different testing approaches are used. 3D airway epithelial tissues allow to mimic exposure conditions in vitro, generates human-relevant toxicology data, allows to elucidate the mode of action of pollutants. Gillian3500 pumps were used to collect the airborne particulate from woodworking and metalworking environments. EpiAirway tissues were used to model half working day (4 h), full working day (8 h), and 3 working day exposures to occupational pollutants. Tissue viability was assessed using an MTT assay. For preliminary assessment, RT-qPCR analyses were performed to analyze the expression of gelsolin, caspase-3, and IL-6. Tissue morphology was assessed by hematoxylin/eosin staining. An effect on the proliferation of lung epithelial cell line A549 was assessed. Acute exposure to workspace pollutants slightly affected tissue viability and did not change the morphology. No inhibiting effect was observed on the proliferation of A549 cells. Preliminary analysis showed that both types of particles suppressed the expression of gelsolin, with the effect of metalworking samples being more pronounced. A slight reduction in caspase-3 expression was observed. Particles from metalworking suppressed IL-6 expression.

## Introduction

Inhalation is a primary exposure route for humans, where the respiratory tract is the target tissue or portal of entry for the systemic circulation for inhaled substances. To evaluate the safety of specific compounds, particles, or mixtures, inhalation exposure studies need to be performed. Inhalation toxicology studies have relied on in vivo and in vitro testing to investigate the toxicity of air pollutants. While animal models for inhalation exposure and toxicology studies are still considered the standard, there is a growing demand for alternative testing strategies^1^*.* Demand for alterative tests is driven by the 3R principle: the replacement, reduction, and refinement of animal usage. Moreover, species-specific differences can have important implications in inhalation toxicity testing humans. Several inhalation toxicity studies in rodents have been proven to lack relevance to humans^2^.

Human-relevant in vitro models allow us to reproduce the distinctive properties of the human airway epithelium. Cell and tissue-based assays are used for inhalation toxicity studies. Immortalized cell lines are also widely used, with the A549 cell line, derived from adenocarcinomic alveolar basal epithelial cells, being the most popular. Other examples of cell lines from the respiratory tract that are commonly used include the human cells lines BEAS-2B and Calu-3 and the rodent cell lines LA-4 and MHS. In past studies, primary cells from human donors, including nasal and bronchial epithelial cells, as well as lung macrophages, were used to examine inhalation toxicology. However, cell-based test systems have several disadvantages. Transformed and immortalized cell lines respond differently from primary cells and may produce irrelevant results. Moreover, monocell cultures that are grown while submerged in a cultivation medium have difficulty closely mimicking in vivo situation^3–5^.

Over the last decade, 3D airway epithelial tissue models have gained acceptance as relevant test systems. Studies have shown that these 3D cell structures represent more physiologically relevant conditions than monocell cultures. To overcome the limitations of animal studies and tests in submerged cell cultures, commercially available airway tissue models have been developed. The MucilAir (Epithelix Corp., Geneva, Switzerland) and EpiAirway (MatTek Corp, Ashland, Massachusetts, USA) systems are examples of commercial airway tissue models that are currently used in research. Both the Mucilair and EpiAirway systems are 3D tissue models reconstituted using primary human respiratory epithelial cells^6,7^. These 3D tissue models are predominantly used in toxicology studies and safety assessments of new materials, including nanomaterials, chemical compounds, drugs, and e-cigaretes^8–11^.

Air pollution with particulate matter is among the largest inducers of negative health effects. Particulate matter is a complex mixture of chemical compounds and biological components. Depending on the source, it varies in its composition, particle size, particle count, and surface area. Fine, ultrafine, and nanoparticles, when inhaled, are able to penetrate deep into the airways and generate negative effects, including the production of oxidative stress and the induction of inflammation. In the airways, the primary targets that particles interact with are epithelial cells and macrophages. However, interactions with other immune cells, endothelial cells, and neurons have also been reported. Apoptosis or programmed cell death is one of the consequences resulting from exposure to air pollutants. Different mechanisms of cytotoxicity and apoptosis induction after exposure to airborne particles have been proposed, including the production of reactive oxygen species, impaired mitochondrial functions, the activation of enzymes involved in apoptosis progression, and inflammation^12–16^.

The goal of the current work was to show the applicability of a tissue based test system for in vitro assessment of the effects of airway exposure to particulate pollutants in a working environment.

## Materials and methods

### Collection and measurement of the particles

Airborne particulates from an 8 h work shift were collected by electrical low-pressure impactor (ELPI+, Dekati) from around the working zones as close to the worker as technically possible: the metalworking industry (M), to assess the processes related to welding (shielded metal arc welding) and milling, and the woodworking industry (W).

The impactor collect the particles on aluminium substrates and classify particles into 14 fractions (Table 1) by their size in the range from 6 nm to 10 μm with a 9.87-lpm sample flow rate using 40-mbar outlet and 1013.3-mbar inlet pressureto analyse occupational air quality based on particle size distribution, number, surface area, and particle mass.

**Table 1:** ELPI+ particles distribution by fractions.

Stage	2	3	4	5	6	7	8
Fraction (μm)	0.0166	0.0274	0.0552	0.0936	0.2620	0.3830	0.6140
Stage	8	9	10	11	12	13	14
Fraction (μm)	0.6140	0.9490	1.6000	2.3900	4.0000	6.6900	9.9200

### Scanning electron microscopy (SEM) and energy dispersive X-ray analysis (EDX)

SEM analysis (NovaNanoSEM 650) by Riga Technical University Faculty of MaterialsScience and Applied Chemistry Institute of Silicate Materials were used to provide the elemental analysis or chemical characterization of the dust particles in a high-vacuum (15 kV) immersion lens mode.

EDX the attachment to high-resolution SEM used to provide the elemental analysis or chemical characterization of the dust particles in an immersion mode. The technique used provided quantitative and spatial analyses of the distribution of chemical elements through mapping (two to three parallel measurements) and point analysis (four to five parallel measurements).

### Effects on proliferation of lung epithelial cells

Human lung epithelial cell line A549 (ATCC, CCL-185) was used to evaluate nanoparticles’ effects on cell proliferation. Cells were seeded on 96-well microplates (Sarstedt) at density of 3500 cells per well in Dulbecco’s modified Eagle’s Medium (Biochrom) supplemented with 100 U/mL penicillin, 100 µg/mL streptomycin (Gibco) and 10% fetal bovine serum (Gibco) and cultivated at 37 °C, 5% CO_2_. After seeding, cells were left to adhere for 24 h. Cell medium was removed and the 100 μL of fresh medium containing nanoparticle suspensions was added. Cells were incubated with nanoparticles for 96 h and monitored during this period with IncuCyte ZOOM live cell imaging system (Essen Biosciences). Using integrated phase contrast microscopy images of cell cultures were taken and recorded once per hour. Kinetics of cell growth was analyzed using the IncuCyte integrated confluence algorithm, where confluence is a surrogate for cell number. Data were obtained from three biological replicates.

### Tissue culture

EpiAirway 3D human respiratory epithelial tissues (AIR-100, surface area 0.6 cm^2^) were purchased from MatTek Corporation (Ashland, MA, USA). The tissues were cultivated at the air–liquid interface in an AIR-100-ASY medium (MatTek) in all exposure experiments. Immediately after arrival, tissue inserts were transferred to 6-well plates containing 1 mL fresh culture medium (AIR-100-ASY, MatTek) and equilibrated for 18 h at 37 °C and 5% CO_2_^17^.

Prior to application of the test samples, the tissue culture inserts were placed on special holders, and 5 mL of fresh basolateral culture medium was added.

### Collection and application of test samples

The airborne particulates from the wood processing and metalworking environments were collected by Gilian 3500 pumps in 5 mL sterile water using suitable receivers (Standard Midget Impingers).

After collection, the samples were freeze-dried (used a lyophilisator Christ Alpha 2-4LD plus). The mass of the freeze-dried particles was determined, and they were resuspended in a sterile phosphate buffered solution (pH 7,4 (hereafter PBS, Biochrom, Germany)) so that one dose to be applied on the tissue culture (e.g., 25 μL) would contain a twofold theoretical concentration of particles in contact with the airway epithelia during a 4-h working period.

To expose the EpiAirway tissues, samples of the particles were collected from the wood processing industry (volume of air pumped was 122.4 L, particle concentration of 1.397 mg/m^3^) and the metalworking industry (volume of air pumped was 106.3 L, particle concentration of 1.018 mg/m^3^). Assuming that the average volume of inhaled air per minute under moderate physical activity is 46 L/min (minute volume limits of 37.3–54.7 L/min, as described in the literature), it is estimated that during 4 h work, the wood worker’s airway epithelium will be exposed to 15.42 mg air-polluting particles, while the metalworking worker will be exposed to 11.24 mg. Since the surface area of the EpiAirway tissue culture is approximately 1.7 × 10^4^ times smaller than the surface area of adult airways, to model exposure over a 4 h period, 0.925 mg polluting particles must be taken from the woodworking industry and 0.674 mg from the metalworking industry. To ensure uniform application of the concentration of the particles, the lyophilised samples were resuspended in a sterile PBS solution (pH 7.4), the exposure to which could be ensured by applying 25 μL of suspension per application to the apical surfaces of the tissues. Before adding them to the tissues to ensure smooth suspension of the particles, dust samples were treated with an ultrasound for 1 min and then vortexed for 1 min.

### Exposure of EpiAirway tissues with polluting particles

Prior to their application, the polluting particle samples were sonicated for 1 min. The apical surface of the EpiAriway tissues was rinsed with PBS to remove mucus. A total of 25 μL particle suspension was added to the apical surface of the tissues. To simulate short term (half working day), full working day, and 3-day exposure, the EpiAirway tissues were treated with samples of the polluting particles for different time periods. The periods were chosen to evaluate the short-term exposure (4 h), exposure to a polluted working environment during a full work shift (8 h), and prolonged (3 working days) exposure to a polluted working environment (72 h). Particles were applied to the tissues and incubated for 4 h, followed by removal of the particle suspension, washing with a PBS solution (pH 7.4), and the addition of a fresh particle suspension. After the second 4-h incubation, the apical surface was washed and incubated for 16 h without the addition of the test samples.

**Figure 1:**
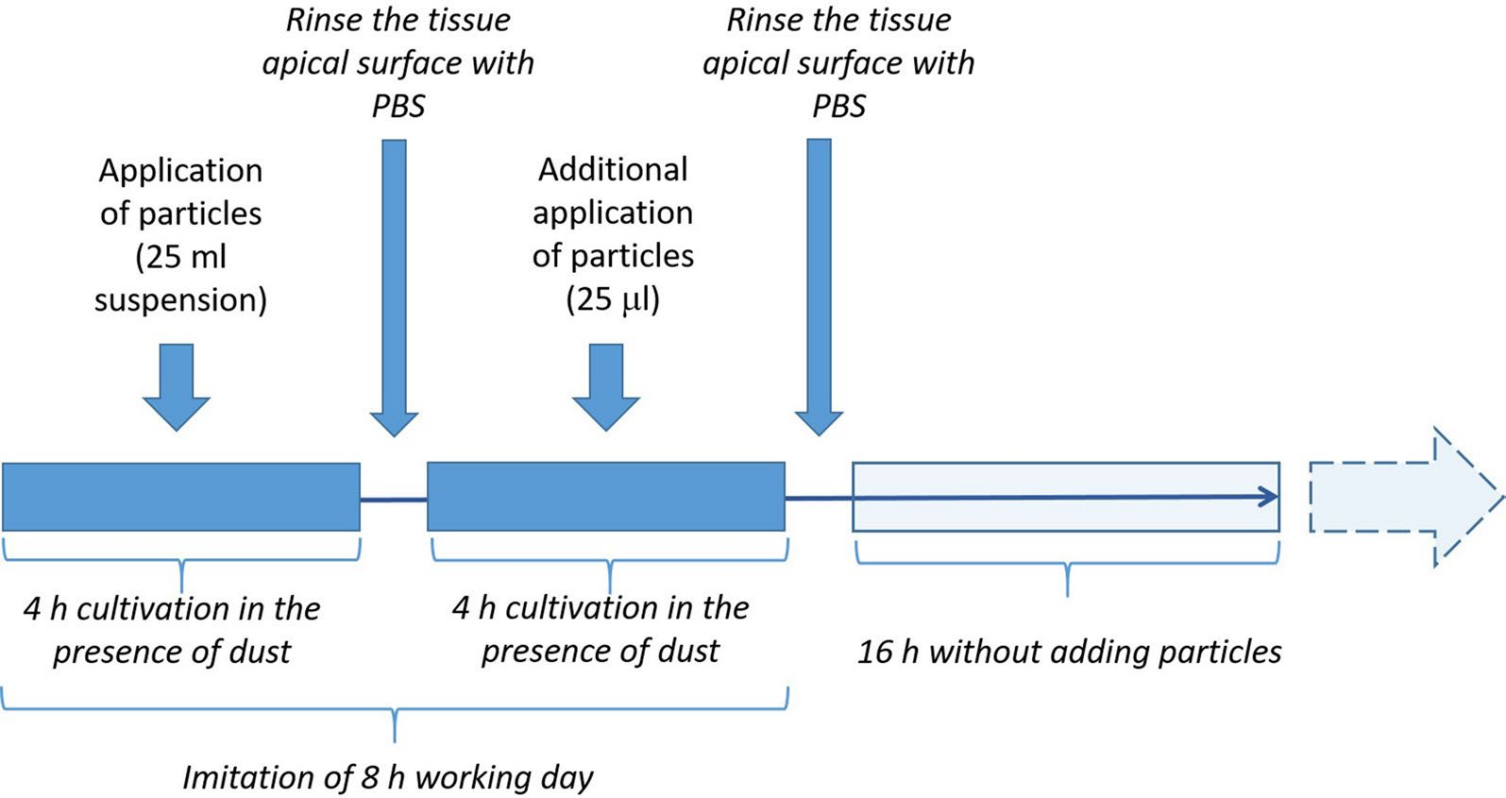
Plan for the in vitro testing of airborne particles in EpiAirway tissue cultures.

To simulate the “daily rest period”, after two 4 h incubation periods with airborne particles, the tissues were incubated overnight (16 h) without the application of polluting particles. The incubation and rest cycles were repeated after 16 h of the rest regime until a 72 h exposure period was reached. All incubations were done at 37 °C and 5% CO_2_ (see the experimental design of the EpiAirway exposure to particle samples in Fig. 1).

Tissues were collected after 4 h, 8 h, and 72 h incubation periods to perform a cell viability analysis, isolate the RNA for gene expression analysis, and perform histological analyses. The tissue culture distribution by group is shown in Table 2.

**Table 2:**
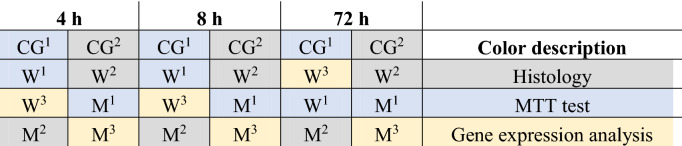
Distribution of tissue cultures by incubation time with the test particle suspensions and their further use for analysis.

*W* woodworking industry, *M* metalworking industry, *C* control group.

^1,2,3^Additional description to colors: 1—MTT test; 2—histology; 3—gene expression analysis.

### MTT tissue viability assay

The 3-(4,5-dimethylthiazol-2-yl)-2,5-diphenyltetrazolium bromide (MTT) and extractant reagents were supplied as a kit (MatTek) and prepared following the supplier’s recommendations. The EpiAirway tissues were transferred to 24-well culture plates containing 300 μL MTT reagent per well and incubated in a 37 °C 5% CO_2_ incubator for 3 h. Following incubation, the EpiAirway tissues were submerged in 2.0 mL MTT extractant solution (MatTek). Tissues were then extracted in the dark at room temperature for 16 h. After extraction, 200 μL of extractant from each tissue was transferred to a clear 96-well plate, and absorbance was measured at 570 nm with the background at 650 nm subtracted. The absorption readings of the control tissue culture (to which no solution containing the pollutants was applied) were taken as 100%, and the viability of the treated tissues was calculated as a percentage of the control level by dividing the sample absorbance values by the control absorbance.

### Assessment of the effects on gene expression in tissue

The relative changes in gene expression were analyzed using a quantitative real-time reverse transcription polymerase chain reaction (qPCR) to assess the effects of air-polluting particles on the expression of three selected genes. EpiAirway tissues were lysed using a Trizol LS reagent (Invitrogen, USA), and RNA was extracted from the cell lysate. Isolated RNA was quantified using Nanodrop ND-100, and 500 ng of RNA was used for the synthesis of complementary DNA (cDNS) using the First Strand cDNA Synthesis Kit (ThermoScientific, USA).

cDNA samples were amplificated using SYBR Green qPCR Master Mix (Thermo Scientific, US) and an ABI Prizm 7300 analyser (Applied Biosystems, USA). Reactions were incubated for 10 min at 95 °C followed by 40 cycles of 15 s at 95 °C and 1 min at 60 °C. The relative changes in gene expression were analyzed by the comparative cycle threshold method (2^−ΔΔCT^) and using the GAPDH gene as in Ref.^18^. The primers used to amplify genes of interest are listed in Table 3.

**Table 3:** Primers used for gene expression analyses.

Gene	Forward primer	Reverse primer	Product length (bp)
GAPDH (NM 001357943.2)	TCCCTGAGCTGAACGGGAAG	GGAGGAGTGGGTGTCGCTGT	218
Gelsolin (NM 001258029.2)	TGGAGGCGACAGCTACATCA	CTCCTTGCCTTGGACCACAC	187
Caspase-3 (NM 004346.4)	GCCTGCCGTGGTACAGAACT	ATGGCACAAAGCGACTGGAT	182
IL-6 (NM 000600.5)	TCGAGCCCACCGGGAACGAA	GCAGGGAAGGCAGCAGGCAA	137

### Histological analysis

For the histological analysis, tissues were fixed in 10% formalin for 30 min at room temperature and rinsed with PBS. The fixed tissue samples were dehydrated using a series of increasing ethanol concentrations cleared with xylene, infiltrated with paraffin, and embedded in paraffin blocks. Tissues were cut using a Leica microtome (sample thickness: 5 μm). Tissue sections were then mounted on a slide, and the prepared slides were kept at 40 °C until dry. The deparaffinization of samples was carried out using xylene/ethanol. The deparaffinized slides were stained with hematoxylin/eosin and viewed using a Leica DM2000 microscope.

## Results and discussion

### Characterization of nanoparticles

**Figure 2:**
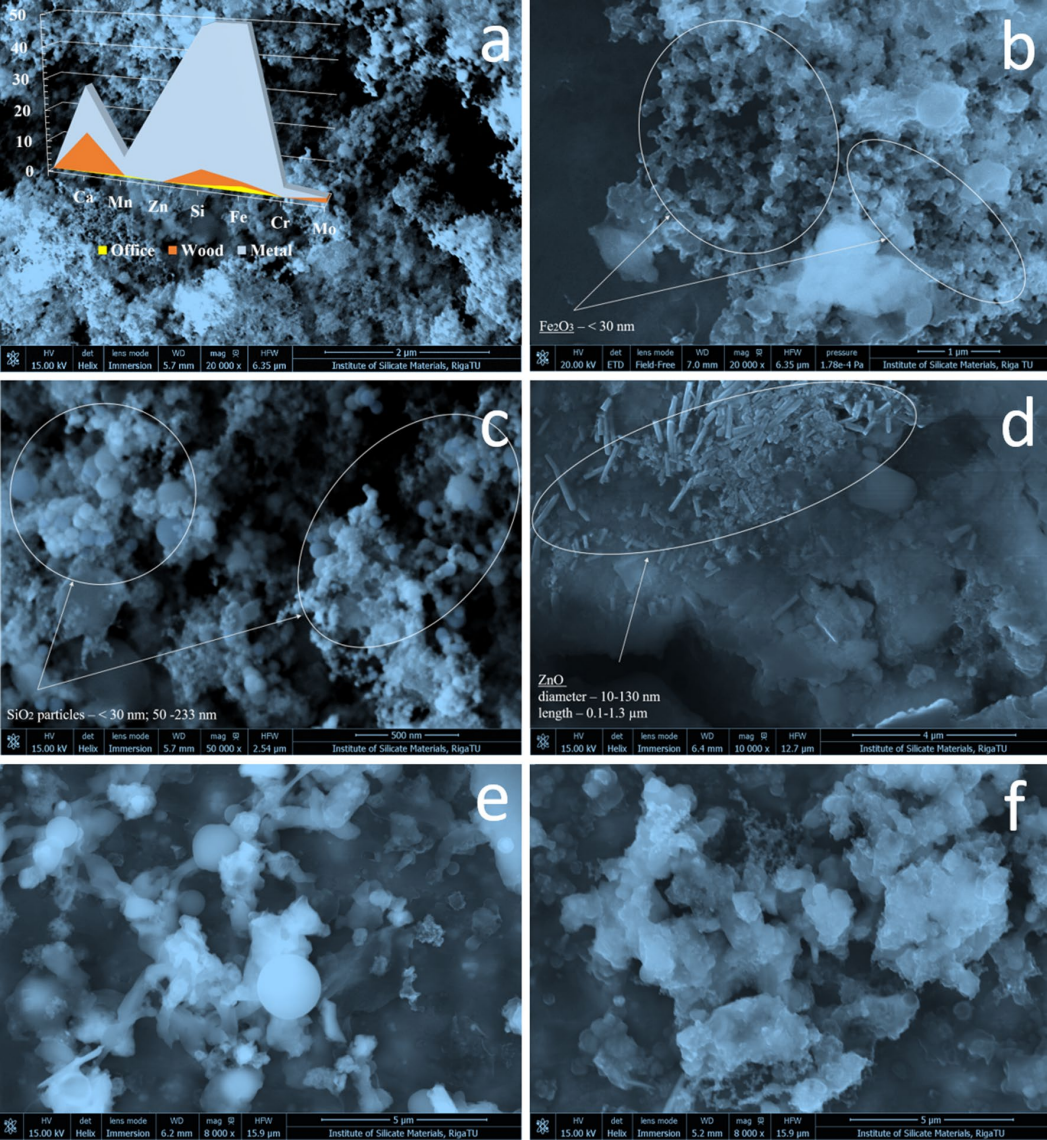
Forms, sizes, and shapes of particles found by SEM: (**a**) particles from ELPI+ Stage 6 (metalworking) and EDX analysis of particle distribution (average from 4 mappings); (**b**) iron oxide nanoparticles; (**c**) silica spheres; (**d**) zinc oxide tubes; (**e**) particles from ELPI+ Stage 9 (woodworking); and (**f**) particles from ELPI+ Stage 6 (woodworking).

The size, shape, quantity, and chemical compositions of nanoparticles were detailed in previous work on the occupational exposure parameters for the characterization of nanoparticulate matter toxicity and a comparison of the biological markers in aerosol-weighed workplaces^19–21^. These studies showed the presence of dust, microscopic, ultramicroscopic (also nanometric (< 100 nm)) containing inorganic and organic dusts, as well as toxic metals like Zn, Si, Fe, and less Ca, Mn, Mo, and Cr. The smallest detected median diameters of the particles (12 nm) and the smallest differences between inhalable dust and the nanosized particle number and mass concentrations were observed in the occupational air of wood industry workplaces (Fig. 2). Furthermore, particle number concentration ratio shows difference between nanosized particles and inhalable dusts. Respectively, the highest nanosized (from Stage1 to 5) particle number concentration ratio were detected in woodworking industry (99.5%), office (92.6%) and metal processing industry (79.5%). The highest number and mass concentration was detected in metalprocessing industry, then woodprocessing and offices (control), respectively^19^. However, shape (spheric, tubes, irregular etc.), quantity (e.g., number and mass concentration) and chemical composition (e.g., SiO_2_, Fe_2_O_3_, ZnO) play a role for further investigation of negative impact to cells and body in general.

The main inorganic elements constituting the particles in order of peak intensity in decreasing order were Fe > Si > Ca > Zn > Cr > Mn and Si > Ca > Fe > Mo for the metal and wood processing groups at all 14 stages, respectively (Fig. 2a). SEM micrographs of particles showed that in air dust are different sized silica spheres (the smallest < 50 nm, average 50–185 nm and larger 208–233 nm, iron oxide with nanoparticles less than 30 nm, and zinc oxide tubes with an average diameter ~ 70 nm (10–130 nm) and 0.1–1.3 microns in length. Calcium in both industries may come from calcium grease that is a high-quality mineral oil based fat, combined with the use of calcium stearates to increase the protection for mechanical parts subjected to high mechanical stress, particularly in high humidity environments with excellent corrosion protection options (Quaker Houghton indicates the 91% reduction in lost parts using calcium oils, www.quakerhoughton.com).

From the results we concluded that metal and wood processing workplaces contain all three types of particulate matter—visible, microscopic, ultramicroscopis and nanoscale (> 10 μm, 0.25–10 μm, < 0.25 μm and 1–100 nm, respectively). Metal processing samples contains much more ultramicroscopic and nanoscale particles right from Stage 2. Wood processing samples contain more microspopic starting from Stage 2 directly. In office samples nanoparticles less than 55 nm generally does not found. The highest concentration of particles in the office environment was iron oxide particles at Stage 8 (< 2%) and may cause by the combustion processes of fuel and diesel fuel or processes of heat energy production in boilers, as well as various production processes that generate dust^19,20^. It is well known, that ZnO nanoparticles (ZnO NPs) entrance and accumulation in the body, and therefore inducing potential toxic effects, is leaded by their size and surface. Pinho et al. study shows data, that higher concentrations of ZnO nanoparticles have a cytotoxic effect to cells, impact to an increase of intracellular Reactive Oxygen Species (ROS) levels, DNA damage, cytoskeleton and nucleoskeleton dynamics alterations, and consequently cell death^22^. In general, the biological effects of zinc oxide particles are caused by zinc ions after intracellular dissolution, by cell-to-particle contacts, and by the uptake of zinc oxide particles into cells^23^. In addition, insoluble ZnO particles predominantly cause local inflammatory effects in the lung. However, ZnO is slightly soluble in water, and animal inhalation studies show, that systemic effects are related to the release of Zn^2+^ ions, because of mass concentration and surface area correlated with the ZnO toxicity rather than the particle concentration^24^. SiO_2_ nanoparticles (NPs) is known to lead pulmonary toxicity. SiO_2_ NPs with primary detected particle size—12 nm (Fig. 2c), caused the accumulation of intracellular Si, the decrease in cell viability, and the decrease in mRNAs expression of surfactant and cause cell apoptosis, as well as the increases in annexin V fluorescence, caspase-3 activity. SiO_2_ NPs exposure increase reactive oxygen species (ROS) production, decrease mitochondrial transmembrane potential^25^.

Metallic nanoparticles (NPs) have potential to cause many chronic pulmonary diseases. Lai et al. study shows, that ZnO nanoparticles, but not Fe_2_O_3_ nanoparticles, induce cell cycle arrest, cell apoptosis, reactive oxygen species (ROS) production, mitochondrial dysfunction and glucose metabolism perturbation, which are responsible for cytotoxicity^26^.

However, Fe_2_O_3_ nanoparticles have toxic effects that are not completely understood yet. The study findings show, that nanoparticles penetrate to the circulation and rapidly reach liver and created serious inflammation in lung and liver tissues. The results show significant enhancement of free radicals and reduction of the GSH in lung tissue. Histological studies show Fe_2_O_3_ nanoparticle treatment of rats caused pulmonary emphysema, interstitial hyperemia and inflammation in lungs^27^.

### Effects on proliferation of lung epithelial cells

**Figure 3:**
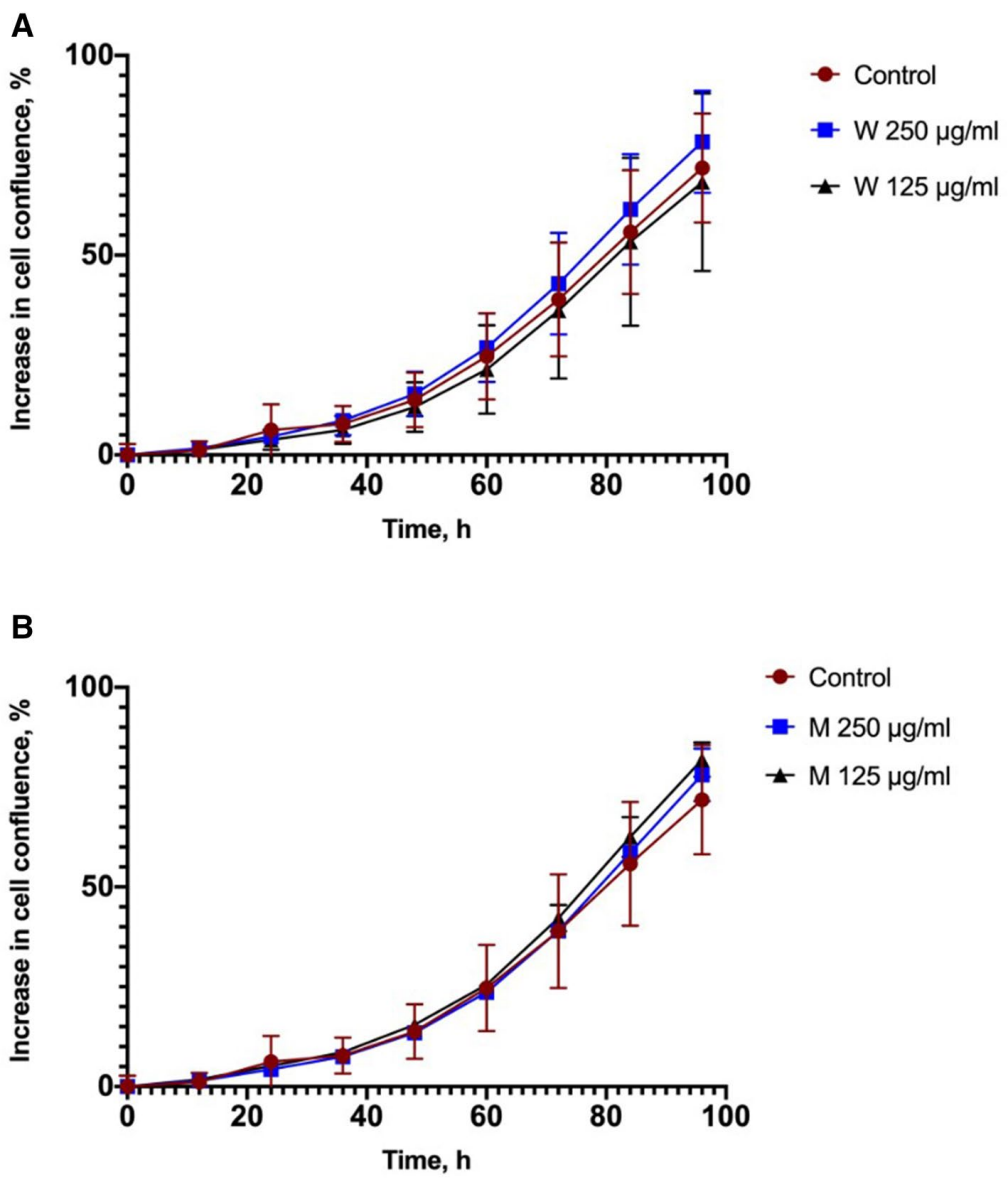
Effects of airborne nanoparticles on proliferation of lung epithelial cell line A549. (**A**) Nanoparticles from woodworking (W) industry, (**B**) nanoparticles from metalworking (M) industry. N = 3.3.3. Impact on the viability of tissues.

Collected nanoparticle samples were tested for their effects on viability and proliferation of lung epithelial cell line A549. Two different nanoparticle concentrations, 125 and 250 µg/mL, from both industries were tested to evaluate if subtoxic concentrations of nanoparticles over 96 h period. Results show that nanoparticles at tested concentrations do not have cytotoxic effect on A549 cells. The rate of proliferation in presence of nanoparticles is similar to that of control (Fig. 3). It has been shown by other authors that airborne heavy metals reduce. A549 viability in a concentration dependent way^28^. Our data are opposite to those observed with PM2.5 particles and urban air particulate pollutants, were viability was significantly reduced already at lowed concentrations than those tested in our study. Various mechanisms of the cytotoxicity have been discussed in other studies, including membrane damages, accumulation of reactive oxygen species, and induction of apoptosis. Polluting particles have been shown to induce expression of proinflammatory cytokines and other signaling molecules^29–31^. Most probably particles tested in our study does not have direct effect on cell viability, but impact on cellular metabolism, expression and secretion of cytokines, growth factors and other signaling molecules would be beneficial to evaluate in further studies. It should be noted that A549 is a carcinomatous cell line that might not fully represent normal airway cell behavior. For further in depth studies either primary cells or 3D airway tissues should be used. In our case we chose EpiAirway tissues to further investigate the effects of particles.

To evaluate the effects of workspace air polluting particles on the viability of the airway epithelia, an MTT assay was used. Tissues were treated apically with particle suspensions to mimic half working day (4 h), full working day (8 h), and three working day (72 h) exposures.

**Figure 4:**
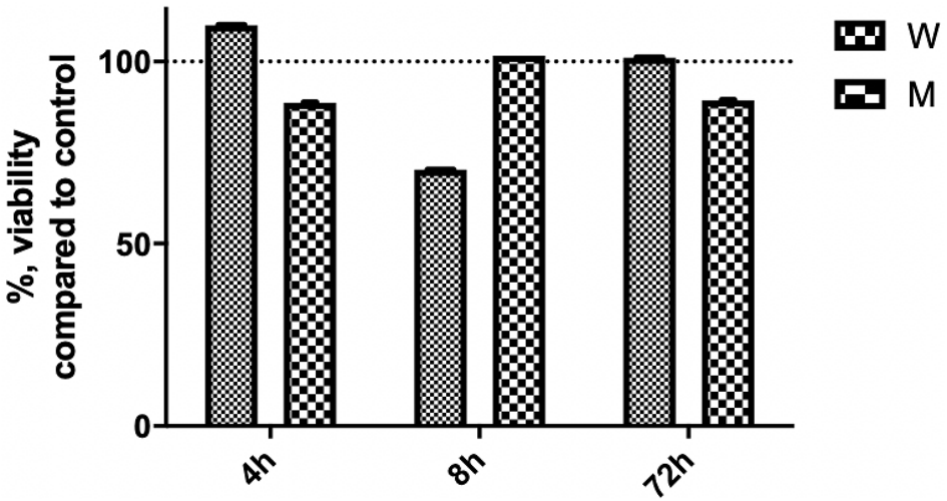
The effect of particles on the viability of the EpiAirway tissue depending on the exposure time. Airborne particles collected from the woodworking industry (W) and metalworking industry (M). The dashed line indicates the control level (n = 3).

Effects on tissue viability varied among exposure times and the sources of the polluting particles (Fig. 4). No statistically significant changes were observed for tissue viability (n = 3). Gene expression analyses did not have biological replicates due to limited availability of tissues (n = 1).

After 4 h exposure to particles from the woodworking industry, a slight increase in tissue viability was still observable. This might be due to the specific stimulation of cell metabolism by particles and the induction of the expression of MTT reducing enzymes. On the contrary, exposure to particles from the metalworking industry slightly reduced tissue viability (by 11.34% compared to the control) within the 4 h incubation period. Differences in the immediate effects after short-term exposure can be explained by the chemical compositions and sizes of the polluting particles from the two workspaces. Different cytotoxicity results have been reported from studies on various nanoparticles, indicating that the effects on the viability of airway tissues depend strongly on the physiochemical properties of the particles^32^. The air polluting particles from the metalworking industry contained higher concentrations of ultramicroscopic and nanometric particles than woodworking. Differences in chemical composition were also observed^19^.

In the case of an 8-h exposure, a pronounced reduction in tissue viability was observed after incubation with particles from the woodworking industry, indicating that negative effects can be expected when exposing the airway epithelium to these types of air-polluting particles for a full working day. Exposure to particles form metalworking for 8 h did not affect tissue viability. This could be explained by the ability of airway epithelial cells to overcome the stress initially caused by the addition of polluting particles. Surprisingly, after exposing tissues to the particles from woodworking for three 8 h cycles, the tissue viability remained unchanged. This indicates that the air-polluting particles from the woodworking company have a more pronounced short-term (acute) cytotoxic effect, but epithelial cells are able to overcome these negative effects over longer exposure. Sixteen hours rest periods between 8 h exposures might be sufficient for the cellular recovery process. For the air samples from the metalworking industry, exposure to polluting particles for three 8 h periods resulted in slightly reduced tissue viability (by 10.64%), indicating the potential long-term negative effects of the airborne metalworking pollutants. In this case, the tissues were able to overcome any temporary adverse effects, but in the longer term, the negative effects accumulated.

Overall, the results indicate that polluting particles from the woodworking and metalworking industries do not induce pronounced acute cytotoxic effects. However, the different results from exposures to samples from these two industries indicate the sensitivity of the tissue-based test system in evaluating acute effects.

### Assessment of the impact on tissue culture structures

**Figure 5:**
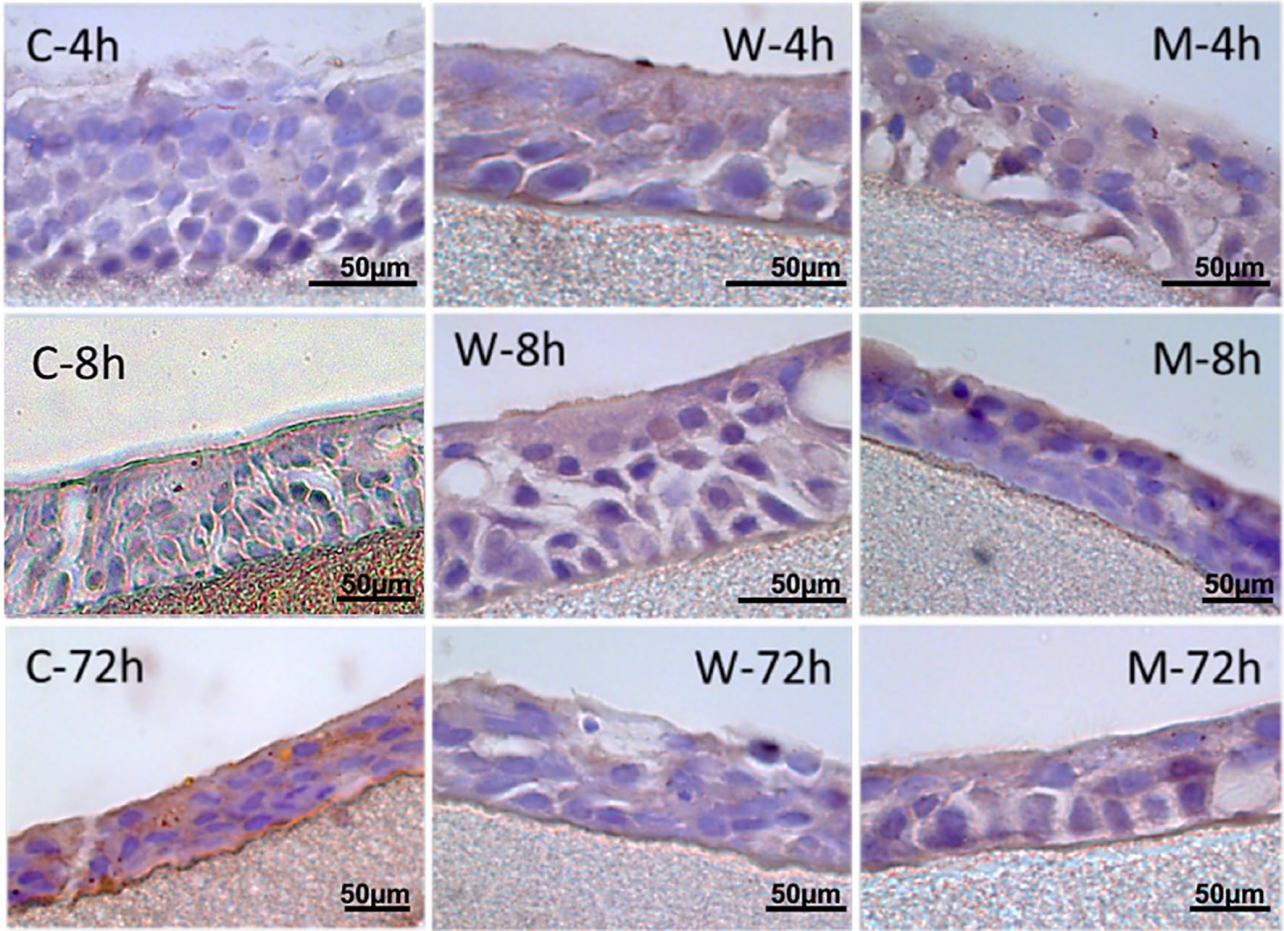
EpiAirway histology and haematosilin coloration under × 1000 magnification: C—control group; W—tissue exposed to the influence of particles collected in the woodworking industry; M—tissue exposed to the influence of particles collected in the metalworking industry.

Histological cuts were prepared and analysed to assess the impact of the pollutants on the tissue’s structure and morphology. All resulting tissue sections showed structures and cells specific to respiratory epithelial tissues (several layers of epithelial cells, the accumulation of mucus in the apical part of the tissue, and the presence of cilia). The histological sections stained with haematosilin are shown in Fig. 5.

Histological sections show that although the negative effects of the tested particles were potentially observed at the cell metabolism (MTT test) and molecular level (gene expression), the morphology of the tissues was not affected.

### Preliminary effects on gene expression

Tissue samples from the simulation of a full working day (8 h) of exposure to particles from the woodworking and metalworking industries were used to isolate the RNA and analyse changes in gene expression.

The preliminary results show that after 8 h incubation with particles, the expression of all three analysed genes was suppressed (Table 4). For both tested types of polluting particles, gelsolin (GSN) gene expression was the most suppressed. Suppression was more pronounced for metalworking particles (34.15% of the control level), although after 8 h exposure, the viability of the tissues was at the control level (n = 3 (technical replicates) for viability, n = 1 for gene expression). For woodworking, gelsolin expression was 50.17% of the control level. Gelsolin is an actin remodeling protein that has been reported to play a role in stress response, exert antioxidative and wound healing activities, and regulate apoptosis in airway tissues. There are contradictory data about the role of gelsolin in response to airway damage, and its role has yet to be confirmed. Some reports indicate decreased levels of gelsolin in lung injury and inflammatory diseases, while others have shown that gelsolin expression is upregulated in the case of airway damage^33–36^. Our previous studies on the expression of gelsolin in employees of the woodworking and metalworking industries agree with these results: Gelsolin gene expression was suppressed compared to office employees^19^. The repression of gelsolin expression after exposure and the agreement with data from industry employees provide a basis for further studies on particles’ effects on gelsolin expression.

**Table 4:** Preliminary results on rrelative changes of gene expression on EpiAirway tissues after 8 h incubation with air-polluting particles.

Origin of particles	Gene of interest		
	Caspase 3	Gelsolin	Interleukine-6
Woodworking	↓ (− 21%)	↓ (− 49.83%)	↓ (− 15.91%)
Metalworking	↓ (− 20.73%)	↓↓ (− 65.85%)	↓↓ (− 53.99%)

↓—Reduction by less than 50% of the control level, ↓↓—reduction by more than 50% of the control level.

Similar changes in the expression of the caspase-3 (CASP-3), a protease involved in apoptosis, were observed for both industries. Particulate pollutants from woodworking reduced expression by 21%, while those from metalworking reduced expression by 20.73%. Some reports in the literature focus on induced caspase-3 gene coding for protease activation and apoptosis in human lung epithelial cells after short-term exposure (24–72 h) to airborne particulate matter^13^. Our preliminary data, however, indicate that airborne particles have a negligible effect on the expression of this apoptotic protein. Further, to elucidate its effects on apoptosis, caspase-3 expression and activity at the protein level should be analyzed. There are differences in the present results compared to the expression of caspase-3 that was observed previously in industry workers; here, expression was slightly suppressed, while previous studies showed increase in woodworking industry employees but suppression in metalworking employees^19^. Changes in caspase-3 expression might be more pronounced after chronic exposure.

The expression of the interleukin 6 (IL-6) gene was slightly reduced (84.09% of the control) compared to the control for the particles from the woodworking industry, while more pronounced suppression (46.01% of the control) of this gene was observed after exposure to particles from the metalworking industry. These differences in expression might be due to different effects resulting from the various sizes and chemical compositions of the particle samples. IL-6 plays a pleiotropic role: It acts both as an inflammatory cytokine and as a mediator in various recovery and regeneration processes; its role in environmental pollutant-induced inflammation has been previously described^37–39^. The expression of IL-6 varies in different parts of the airways^38^. The suppression of IL-6 may, therefore, have a positive effect, indicating the tissue’s ability to suppress excessive inflammation in response to particles. It might also have a negative impact on tissue recovery. These results are opposite to those from studies of nanomaterials, where the expression of IL-6 was increased in a 3D airway model after acute exposure^40^; the results are also different from the expression levels of industry workers. Previous studies on IL-6 levels showed that in woodworking employees, IL-6 expression is increased, but in metalworking employees, the expression levels were similar to those of office workers^19^. Differences between various studies and our results could be explained by exposure times. Analysis of protein levels were not performed in this study, but it would enrich research data regarding to tissue responses explanation better and could be provided during next research stages.

## Conclusions

Our study confirms that 3D airway epithelial tissues can be used as a relevant system to model exposure to workspace pollutants and evaluate their effects. The polluting particles tested in this study show minor effects on viability of A549 cells and EpiAirway tissues and tissue morphology after a short period of exposure. Preliminary gene expression analysis showed that particles from different working environments affect the expression of gelsolin and IL-6 genes differently. It should be emphasized that the most suppressed gelsolin gene was also reduced among the employees from both industries in our previous studies, highlighting this protein as a potential marker for occupational pollutant effects on airway tissues.

## Data Availability

All data analyzed within this study are included in the manuscript.

## References

[1] Zavala J, O’Brien B, Lichtveld K, Sexton KG, Rusyn I, Jaspers I, Vizuete W (2017). Assessment of biological responses of EpiAirway 3-D cell constructs vs. A549 cells for determining toxicity of ambient air pollution. Inhal. Toxicol..

[2] Mowat V, Alexander DJ, Pilling AM (2017). A comparison of rodent and nonrodent laryngeal and tracheal bifurcation sensitivities in inhalation toxicity studies and their relevance for human exposure. Toxicol. Pathol..

[3] Upadhyay S, Palmberg L (2018). Air–Liquid Interface: Relevant In Vitro Models for Investigating Air Pollutant-Induced Pulmonary Toxicity.

[4] Hiemstra PS, Grootaers G, van der Does AM, Krul CAM, Kooter IM (2018). Human lung epithelial cell cultures for analysis of inhaled toxicants: Lessons learned and future directions. Toxicol. In Vitro.

[5] Zavala J, O'Brien B, Lichtveld K, Sexton KG, Rusyn I, Jaspers I, Vizuete W (2016). Assessment of biological responses of EpiAirway 3-D cell constructs versus A549 cells for determining toxicity of ambient air pollution. Inhal. Toxicol..

[6] Rothen-Rutishauser B, Blank F, Mühlfeld C, Gehr P (2008). In vitro models of the human epithelial airway barrier to study the toxic potential of particulate matter. Expert Opin. Drug Metab. Toxicol..

[7] Zavala J, Freedman AN, Szilagyi JT, Jaspers I, Wambaugh JF, Higuchi M, Rager JE (2020). New approach methods to evaluate health risks of air pollutants: Critical design considerations for in vitro exposure testing. Int. J. Environ. Res. Public Health.

[8] Fields W, Maione A, Keyser B, Bombick B (2017). Characterization and application of the vitrocell VC1 smoke exposure system and 3D EpiAirway models for toxicological and e-cigarette evaluations. Appl. Vitro Toxicol..

[9] Czekala L, Simms L, Stevenson M, Tschierske N, Maione AG, Walele T (2019). Toxicological comparison of cigarette smoke and e-cigarette aerosol using a 3D in vitro human respiratory model. Regul. Toxicol. Pharmacol..

[10] Jackson GR, Maione AG, Klausner M, Hayden PJ (2018). Prevalidation of an acute inhalation toxicity test using the epiairway in vitro human airway model. Appl. In Vitro Toxicol..

[11] Chapman KL, Holzgrefe H, Black LE, Brown M, Chellman G, Copeman C, Couch J, Creton S, Gehen S, Hoberman A (2013). Pharmaceutical toxicology: Designing studies to reduce animal use, while maximizing human translation. Regul. Toxicol. Pharmacol..

[12] Andreau K, Leroux M, Bouharrour A (2012). Health and cellular impacts of air pollutants: From cytoprotection to cytotoxicity. Biochem. Res. Int..

[13] Dagher Z, Garcon G, Billet S, Gosset P, Ledoux F, Courcot D, Aboukais A, Shirali P (2006). Activation of different pathways of apoptosis by air pollution particulate matter (PM2.5) in human epithelial lung cells (L132) in culture. Toxicology.

[14] Ovrevik J, Refsnes M, Leg M, Holme JA, Schwarze PE (2015). Activation of proinflammatory responses in cells of the airway mucosa by particulate matter: Oxidant- and non-oxidant mediated triggering mechanisms. Biomolecules.

[15] Pardo M, Qiu X, Zimmermann R, Rudich Y (2020). Particulate matter toxicity is Nrf2 and mitochondria dependent: The roles of metals and polycyclic aromatic hydrocarbons. Chem. Res. Toxicol..

[16] Peixoto MS, de OliveiraGalvão MF, Batistuzzo de Medeiros SR (2017). Cell death pathways of particulate matter toxicity. Chemosphere.

[17] Revoir WH (1997). Respiratory Protection Handbook.

[18] Yacobi NR, Fazllolahi F, Kim YH, Sipos A, Borok Z, Kim KJ, Crandall ED (2011). Nanomaterial interactions with and trafficking across the lung alveolar epithelial barrier: Implications for health effects of air-pollution particles. Air Qual. Atmos. Health.

[19] Pavlovska I, Martinsone Z, Vanadzins I, Martinsone I, Seile A, Sudmalis P (2016). Occupational Exposure Parameters for Characterization of Nanoparticulate Matter Toxicity: Metal Versus Wood Processing.

[20] Pavlovska I, Martinsone Ž, Ramata-Stunda A, Vanadzins I, Martinsone I, Seile A (2019). Comparison of biological markers in aerosol-weighed workplaces. J. Nanopart. Res..

[21] Livak KJ, Schmittgen TD (2001). Analysis of relative gene expression data using real-time quantitative PCR and the 2(-Delta Delta C(T)) method. Methods.

[22] Pinho AR, Martins F, Costa MEV, Senos AMR, da Cruz e Silva AB, Pereira ML, Rebelo S (2020). In vitro cytotoxicity effects of zinc oxide nanoparticles on spermatogonia cells. Cells.

[23] Olejnik M, Kersting M, Rosenkranz N (2021). Cell-biological effects of zinc oxide spheres and rods from the nano- to the microscale at sub-toxic levels. Cell Biol. Toxicol..

[24] Ho M, Wu KY, Chein HM, Chen LC, Cheng TJ (2011). Pulmonary toxicity of inhaled nanoscale and fine zinc oxide particles: Mass and surface area as an exposure metric. Inhal. Toxicol..

[25] Lee KI, Su CC, Fang KM (2020). Ultrafine silicon dioxide nanoparticles cause lung epithelial cells apoptosis via oxidative stress-activated PI3K/Akt-mediated mitochondria- and endoplasmic reticulum stress-dependent signaling pathways. Sci. Rep..

[26] Lai X, Wei Y, Zhao H, Chen S, Bu X, Lu F, Qu D, Yao L, Zheng J, Zhang J (2015). The effect of Fe2O3 and ZnO nanoparticles on cytotoxicity and glucose metabolism in lung epithelial cells. J. Appl. Toxicol..

[27] Sadeghi L, Yousefi Babadi V, Espanani HR (2015). Toxic effects of the Fe_2_O_3_ nanoparticles on the liver and lung tissue. Bratisl. Lek. Listy..

[28] Choi Y, Park K, Kim I, Kim SD (2016). Combined toxic effect of airborne heavy metals on human lung cell line A549. Environ. Geochem. Health.

[29] Zhang Y, Darland D, He Y, Yang L, Dong X, Chang Y (2018). reduction of PM2.5 toxicity on human alveolar epithelial cells A549 by tea polyphenols. J. Food Biochem..

[30] Vuong NQ, Breznan D, Goegan P (2017). In vitro toxicoproteomic analysis of A549 human lung epithelial cells exposed to urban air particulate matter and its water-soluble and insoluble fractions. Part. Fibre Toxicol..

[31] Mazuryk O, Stochel G, Brindell M (2020). Variations in reactive oxygen species generation by urban airborne particulate matter in lung epithelial cells-impact of inorganic fraction. Front. Chem..

[32] Maniatis NA, Harokopos V, Thanassopoulou A, Oikonomou N, Mersinias V, Witke W, Orfanos SE, Armaganidis A, Roussos C, Kotanidou A, Aidinis V (2009). A critical role for gelsolin in ventilator-induced lung injury. Am. J. Respir. Cell. Mol. Biol..

[33] Gupta AK, Chopra BS, Vaid B, Sagar A, Raut S, Badmalia MD, Khatri N (2019). Protective effects of gelsolin in acute pulmonary thromboembolism and thrombosis in the carotid artery of mice. PLoS ONE.

[34] Vaid B, Chopra BS, Raut S, Sagar A, Badmalia MD, Khatri N (2020). Antioxidant and wound healing property of gelsolin. Oxid. Med. Cell. Longev..

[35] Koya RC, Fujita H, Shimizu S, Ohtsu M, Takimoto M, Tsujimoto Y, Kuzumaki N (2000). Gelsolin inhibits apoptosis by blocking mitochondrial membrane potential loss and cytochrome c release. J. Biol. Chem..

[36] Kobayashi T, Tanaka K, Fujita T, Umezawa H, Amano H, Yoshioka K, Naito Y, Hatano M, Kimura S, Tatsumi K, Kasuya Y (2015). Bidirectional role of IL-6 signal in pathogenesis of lung fibrosis. Respir. Res..

[37] Rincon M, Irvin CG (2012). Role of IL-6 in asthma and other inflammatory pulmonary diseases. Int. J. Biol. Sci..

[38] Paplińska-Goryca M, Nejman-Gryz P, Chazan R, Grubek-Jaworska H (2013). The expression of the eotaxins IL-6 and CXCL8 in human epithelial cells from various levels of the respiratory tract. Cell Mol. Biol. Lett..

[39] Yu M, Zheng X, Witschi H, Pinkerton KE (2002). The role of interleukin-6 in pulmonary inflammation and injury induced by exposure to environmental air pollutants. Toxicol. Sci..

[40] Skuland T, Låg M, Gutleb AC (2020). Pro-inflammatory effects of crystalline- and nano-sized non-crystalline silica particles in a 3D alveolar model. Part Fibre Toxicol..

